# An Intracortical Implantable Brain-Computer Interface for Telemetric Real-Time Recording and Manipulation of Neuronal Circuits for Closed-Loop Intervention

**DOI:** 10.3389/fnhum.2021.618626

**Published:** 2021-02-03

**Authors:** Hamed Zaer, Ashlesha Deshmukh, Dariusz Orlowski, Wei Fan, Pierre-Hugues Prouvot, Andreas Nørgaard Glud, Morten Bjørn Jensen, Esben Schjødt Worm, Slávka Lukacova, Trine Werenberg Mikkelsen, Lise Moberg Fitting, John R. Adler, M. Bret Schneider, Martin Snejbjerg Jensen, Quanhai Fu, Vinson Go, James Morizio, Jens Christian Hedemann Sørensen, Albrecht Stroh

**Affiliations:** ^1^Department of Neurosurgery, Center for Experimental Neuroscience (CENSE), Aarhus University Hospital, Aarhus, Denmark; ^2^Department of Clinical Medicine, Aarhus University, Aarhus, Denmark; ^3^Department of Electrical and Computer Engineering, Pratt School of Engineering, Duke University, Durham, NC, United States; ^4^Department of Biomedical Engineering, Pratt School of Engineering, Duke University, Durham, NC, United States; ^5^Leibniz Institute for Resilience Research, Mainz, Germany; ^6^Department of Oncology, Radiation Therapy, and Clinical Medicine, Aarhus University Hospital, Aarhus University, Aarhus, Denmark; ^7^Zap Surgical Systems, Inc., San Carlos, CA, United States; ^8^Department of Neurosurgery, Stanford University School of Medicine, Stanford, CA, United States; ^9^Department of Psychiatry and Behavioral Sciences, Stanford University School of Medicine, Stanford, CA, United States; ^10^Department of Nuclear Medicine and PET Center, Institute of Clinical Medicine, Aarhus University and Hospital, Aarhus, Denmark; ^11^Institute of Pathophysiology, University Medical Center of the Johannes Gutenberg University Mainz, Mainz, Germany

**Keywords:** brain-machine (computer) interface, electrophysiology, neuromodulation, animal model, EEG, stereotactic radiosurgery, Göttingen minipig, closed-loop

## Abstract

Recording and manipulating neuronal ensemble activity is a key requirement in advanced neuromodulatory and behavior studies. Devices capable of both recording and manipulating neuronal activity brain-computer interfaces (BCIs) should ideally operate un-tethered and allow chronic longitudinal manipulations in the freely moving animal. In this study, we designed a new intracortical BCI feasible of telemetric recording and stimulating local gray and white matter of visual neural circuit after irradiation exposure. To increase the translational reliance, we put forward a Göttingen minipig model. The animal was stereotactically irradiated at the level of the visual cortex upon defining the target by a fused cerebral MRI and CT scan. A fully implantable neural telemetry system consisting of a 64 channel intracortical multielectrode array, a telemetry capsule, and an inductive rechargeable battery was then implanted into the visual cortex to record and manipulate local field potentials, and multi-unit activity. We achieved a 3-month stability of the functionality of the un-tethered BCI in terms of telemetric radio-communication, inductive battery charging, and device biocompatibility for 3 months. Finally, we could reliably record the local signature of sub- and suprathreshold neuronal activity in the visual cortex with high bandwidth without complications. The ability to wireless induction charging combined with the entirely implantable design, the rather high recording bandwidth, and the ability to record and stimulate simultaneously put forward a wireless BCI capable of long-term un-tethered real-time communication for causal preclinical circuit-based closed-loop interventions.

## Introduction

The recent advances in recording and manipulation techniques, particularly in preclinical animal models, significantly furthered our understanding of the neuronal circuit functions underlying complex behaviors. It becomes now apparent, that complex and multifactorial disorders of the CNS such as depression could be classified as neural circuit disorders (Insel et al., 2010). Yet, for probing the contributory role of distinct components of a neuronal circuitry to a given (dys)-function, a causal, real-time intervention is mandatory. Ideally, a current neuronal activity signature should inform the manipulation schemes in a closed-loop fashion, in an unrestrained, unperturbed subject. Integrated devices capable of interfacing with the external experimenter are termed brain-computer interfaces (BCI) or Brain-machine interfaces (BMI). The first requirement for BCI is a dense recording of the current activity state of the given circuitry. For that, superficial, non-invasive Electroencephalography (EEG) is already well established in the clinical diagnosis of neurological disorders (Lebedev and Nicolelis, 2006). In the framework of BCI, EEG represents the recording module of BCIs enabling the patient to drive an electronic spelling device employing slow cortical potentials (SCPs) (Birbaumer et al., 1999). Also, locked-in amyotrophic lateral sclerosis (ALS) patients can learn to control virtual keyboards via slow cortical potentials or oscillatory EEG components as the input signals for the BCIs (Pfurtscheller et al., 2003; Hinterberger et al., 2005). Until recently, the precision of the non-invasive EEG-based interfaces was limited by a rather low spatial resolution (Srinivasan, 1999), poor signal-to-noise ratio (Bang et al., 2013), and low transfer rate (Wolpaw et al., 2002). Particularly in preclinical animal studies, hard-wired devices restrict the degrees of freedom for behavior studies. However, the newer generation of minimally/non-invasive wearable EEG-based seizure detection devices utilizing closed-loop warning systems, and non-EEG-based devices employing accelerometer (ACM), Electromyography (EMG), etc., have incredibly assisted the clinical management of epileptic disorders (Borujeny et al., 2013; Ramgopal et al., 2014; Van de Vel et al., 2016). Still, long-term monitoring of brain activity via scalp EEG devices has some shortcomings which could be anticipated to be addressed by sub-scalp EEG devices (Duun-Henriksen et al., 2020).

Invasive methods are subclassified into intracranial EEG (ECoG and stereo-EEG) or intracortical and intraparenchymal micro-arrays where penetrating electrodes are employed to target deeper regions including the limbic system (Parvizi and Kastner, 2018). Such methods provide recordings with broader temporal bandwidth up to 500 Hz (Staba et al., 2002; Butterfield et al., 2007), better spatial resolution, and typically a higher amplitude, as the electrodes are closer to the neural tissue (Lebedev and Nicolelis, 2006; Ball et al., 2009). The iEEG recordings are less affected by electric potentials caused by e.g., the cranial muscles or eye movements (Mak and Wolpaw, 2009). Indeed, ECoG is used for BCIs implemented in neural motor prosthesis devices for paralyzed patients e.g., fully implanted BCI in locked-in ALS patients (Vansteensel et al., 2016). Invasive methods might therefore be more suitable for BCIs due to the higher spatial precision of recording (tenths of millimeters) and lesser need for user training than scalp EEG based systems (Wolpaw et al., 2002; Leuthardt et al., 2004; Lebedev and Nicolelis, 2006; Schalk and Leuthardt, 2011). Long-term clinical usage of invasive BCIs employing micro-arrays is, however, limited by complications related to the implantation surgery and long-term recording instability due to signal degradation of the impedances of the recording sites caused by encapsulation and displacement of electrodes (Shain et al., 2003; Schalk et al., 2007). On the technical side, the low signal transfer rate (below 10 megabits/s) limits the large-scale investigation of brain activity signatures with adequate resolution. A hard-wired interface communication with an extra-corporal remote terminal constrains the movement of the patient/experimental subject (Liu et al., 2018; Zaer et al., 2020). Partly wireless systems allow rather unrestraint movements of the patients, but, still need to be constantly inductively powered when in use (Guenther et al., 2009; Liu et al., 2018).

Another important example of neural interfacing devices as deep brain stimulation (DBS) devices are not classically described as BCI devices. However, the newer generation of DBS units combined with the concept of Bi-directional BCI, for instance in Parkinson’s patients, investigates the possibility of stimulation by utilizing the sensing electrodes according to the intermittent nature of the disease symptoms (Angeles et al., 2016).

In terms of achieving the real-time acquisition of the current local neuronal activity state, the use of microelectrode arrays has gained momentum. Recent advances in array design and fabrication allow for the production of ideally tailored multichannel probes according to the geometry of the targeted brain region, in both preclinical and even clinical studies. Depending on the coating, diameter, and distance of the individual electrode sites, not only the local field potential can be assessed, but also the recording of multi- and single-unit (MU and SU) activity is attainable. Spike sorting and mapping enable the identification of the excitatory vs. inhibitory neurons can be identified (Yang et al., 2017). Of particular importance is the high spatial resolution given by the dense array of individual electrode sites. This enables the recording, identification, and replay of complex patterns of local neuronal ensemble activities, due to the ability to use various sites for stimulation. Multielectrode arrays, therefore, may represent a suitable solution for real-time closed-loop applications.

In this study, we designed a new fully implantable invasive intra-parenchymal BCI for electrophysiological telecommunication in a large animal model using the Göttingen minipig to at least partially overcome the aforementioned current limitations due to hard-wired charging systems and rather low bandwidth. These animals have a sufficiently large brain for testing the human-sized implants, in contrast to the small rodent brain (Gierthmuehlen et al., 2014). The physiology, internal anatomy, and even genome of the Göttingen minipigs are reasonably similar to humans in comparison to rodents (Dolezalova et al., 2014; Sjöstedt et al., 2020). Moreover, Göttingen minipigs are also suited for chronic studies as they grow slowly to a maximum of 35 kg and allow biocompatibility assessment of the implanted materials over long periods providing an accessible large animal model for various preclinical translational study (Sørensen et al., 2011; Bro et al., 2012).

To validate the function and quality of the device we used visual cortical lesions made by ablative radiosurgery. While ablative radiosurgery has been used for decades, relatively recently, studies suggested a neuromodulatory effect of sub-necrotic doses on irradiated neurons (Schneider et al., 2010). For instance, radiosurgery of epileptogenic arteriovenous malformations (AVM) in functional areas has shown cessation or remission of epileptic attacks, unexpectedly prior to fully obliteration of AVM and visible changes in MRI (Steiner et al., 1992). This gap between appearing MRI or histological changes and the clinical effect of irradiation raised the idea of the potential neuromodulatory effect of sublethal radiodoses (Regis et al., 1995, 1999). Irradiating a specifically targeted area within the brain circuits with sublethal doses (“radio-modulation”) is postulated to alter the function of the circuit as a whole (Schneider et al., 2010). The effect of radiation is suggested to be depending on the radiation dose, type, and volume of the targeted brain tissue (Regis, 2014). Below the necrotic radiodoses, there may still be alterations, not visible in anatomical imaging; therefore, evaluation of post-irradiation functional changes in neuronal circuits is mandatory for radiation dose-adjustment to obtain neuromodulatory effects without necrosis. Here, we chose the primary visual cortex as the implantation site. While certainly the primary visual cortex is tasked with the representation and computation of visual afferents, it becomes apparent, that even primary sensory cortical areas go beyond the functions for which they are named. Recent pieces of evidence suggest, that primary cortical networks exhibit complex dysregulations mirroring behavioral states in neuronal disorders originating in distant regions (Iaccarino et al., 2016; Arnoux et al., 2018; Ellwardt et al., 2018). Moreover, the visual cortex (V1) in Göttingen minipigs is surgically easily accessible and big enough to facilitate the process of radiosurgical targeting and implantation of invasive electrodes. By choosing the visual cortex, we could simultaneously achieve different goals. First, verification of the functionality of this BCI. Second, piloting the stereotactic radiosurgical approach on the very thin cortical layer of the visual cortex (2–3 mm). Third, assessing the feasibility of observing radio-neuromodulatory changes by this system. In this study, as a part of the larger project on evaluation of the neuromodulatory effect of ionizing radiation, we aimed to design a BCI to survey the real-time electrophysiological events after stereotactic radiosurgery in the visual cortex.

## Materials and Methods

One female Göttingen minipig, age 6 months, weight 15 kg, was used for this proof of concept study. The Danish Animal Experiments Inspectorate (2016-15-0201-01103) approved this study in compliance with the ARRIVE guidelines and the 2010/63/EU directive for animal experiments. After a month of acclimatization in a standardized research environment facility, the animal underwent Magnetic Resonance Imaging (MRI) of the brain as a baseline and to define the target area for the stereotactic radiosurgery and then implantation of the device as described below in each corresponding subsection.

### Radiotherapy Treatment Planning and Treatment Delivery

**FIGURE 1:**
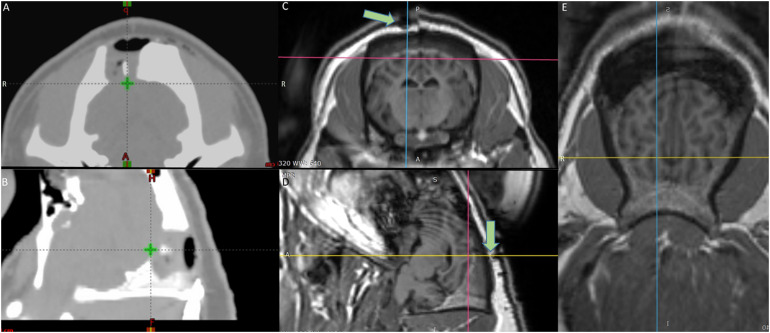
Target definition by the fused MRI/CT **(A)** axial and **(B)** sagittal view CT scans of the radiotherapy planning system – The green cross shows the target area on the visual cortex (V1) irradiated with 100 Gy. **(C)** Axial **(D)** Sagittal **(E)** Horizontal view MRI images of the brain – the crosshair shows the target area – Green arrows in **(C,D)** point at the fiducial marker on top of the irradiated area.

Stereotactic radiosurgery treatment planning was based on fused CT and MR image data sets. The planning CTs were acquired with 1.5 mm slice thickness (Brilliance Big Bore CT, Phillips, Amsterdam, Netherlands) (Figures 1A,B). The T1 MR scans were acquired with a 1.0 mm slice thickness (Figures 1C–E). A point target was defined in the fused CT/MR data sets. Treatment was planned using the Eclipse treatment planning system (Eclipse 13.7, Varian Medical Systems, Palo Alto, CA, United States) and based on a Truebeam linear accelerator equipped with an HD 120 multileaf collimator (MLC) of 2.5 mm leaf widths. The treatment plan consisted of 14 beams (6MV; dose rate 600 MU/min) using a non-coplanar beam arrangement with MLC defined apertures centered at the target. Aperture sizes were quadratic 5 × 5 mm with a calculated target dose of 100 Gy normalized to the defined point target (0.03 cm^3^ receiving 80 Gy or above). The dose calculated by the Eclipse treatment planning system (Acuros v. 13.7.14 dose calculation algorithm with calculation grid size 0.1 cm) was corrected with small-field dosimetric factors obtained from measurements with a diamond detector [Natural diamond detector, type 600003 (PTW Freiburg GmBH)] in a water phantom. Localization of the irradiation target was obtained by fusing a pre-treatment 1.5 mm slice thickness kV cone-beam CT (CBCT) scan with the planning CT, using the onboard imaging system of the Truebeam accelerator. Couch corrections were performed according to the CBCT with 6 degrees of freedom (translational, rotation, pitch, and roll). A verification CBCT was taken after couch correction (before treatment) to assess if the animal had moved during the correction. An additional CBCT was taken post-treatment to verify intra-fractional positional stability.

### Surgical Procedure

**FIGURE 2:**
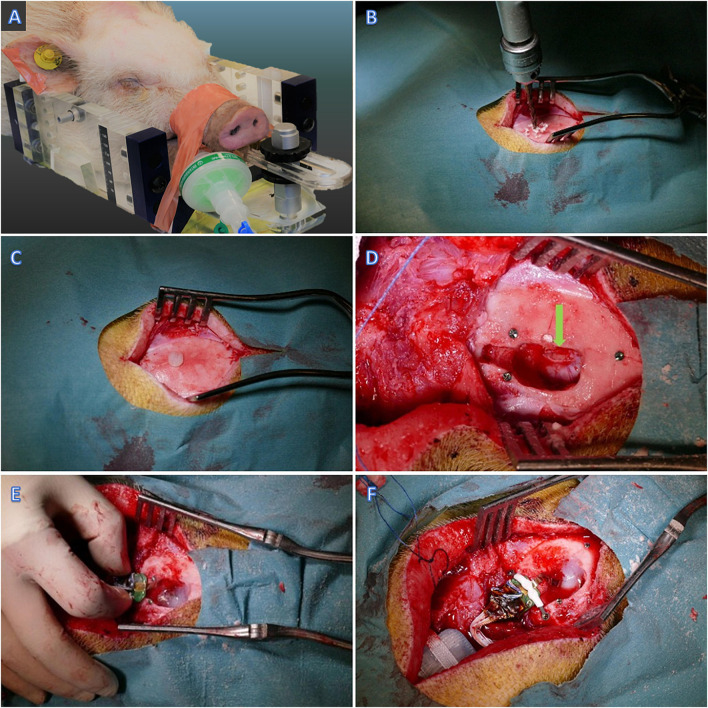
Implantation surgery. **(A)** The minipig fixated in the MRI compatible head frame. **(B)** Drilling the skull posterior to Bregma **(C,D)** Plastic and titanium fiducial markers. **(D)** Visual cortex exposure – pointed by green arrow. **(E)** Insertion of the probe in the visual cortex and fixation with Fibrin Sealant. **(F)** Fixation of the Omnetics connector to the skull bone.

The animal was sedated with an intramuscular injection of Midazolam (0.8 mg/kg)/Ketamine (20 mg/kg) mixture as premedication. An ear vein was catheterized (21G venflon) and anesthesia was induced with a second IV injection of Midazolam/S-ketamine mixture. Shortly afterward, the minipig was intubated (4.5 mm cuffed tube) and anesthesia was maintained using approximately 2% isoflurane through mechanical ventilation and a mixture of 50% O2. Intravenous injection of propofol (20 ml/h) was used during transportation for the imaging procedures. The pig was fixated prone in an MRI-compatible localizer box (Figure 2A; Bjarkam et al., 2004). A mid-sagittal skin incision was made, exposing the skull. The periosteum was removed, and a small drill hole was made posterior to the bregma (Figure 2B). A plastic screw containing copper sulfate was placed in the drill hole, providing a fixed point for the estimation of stereotaxic coordinates (Glud et al., 2017). Three titanium screws were also put in the skull anterior, posterior, and lateral to the fiducial marker as landmarks for more accuracy during the implantation surgery (Figures 2C,D). Preoperative MRI of the animal fixated in the localizer box was carried out using a 3D T1-weighted MRI brain scan (3.0 T Siemens Skyra). The visual cortex was visualized according to the online Brain Atlas of Göttingen minipig (Orlowski et al., 2019). The location of the target (visual cortex) in relation to the fiducial marker was calculated. The cranium was opened by surgical drill and the visual cortex was exposed after opening the dura (Figure 2D). The probe was placed using microsurgical forceps directly into the visual cortex and then the flexible outcoming wire was fixed by fibrin sealant (TISSEEL^®^, Baxter, United States) above the dura (Figure 2E). The Omnetics connector afterward was attached to the skull bone with two titanium screws and an interconnecting plastic bar (Figure 2F). The rest of the flexible wire between the probe and the Omnetics connector then was secured with Bioglue^®^, (CryoLife, United States). Three of the ground wires were put in the muscles and one underneath the titanium screw and thereby fixated to the skull bone. The battery and communicating capsule was placed into the subcutaneous pocket in the posterior neck region and sutured to the muscles with a holding Dacron ribbon (Figure 2F). Then, the surgical wound was closed. The animal received prophylactic antibiotic [Benzyl Penicillin procaine 30,000 IE/kg (Penovet^®^, Boehringer Ingelheim, Denmark)] and analgesic [Meloxicam 1.5 mg/kg (Metacam^®^, Boehringer Ingelheim, Denmark)] both once a day for 5 days.

### Design of the Telemetric Brain-Computer Interface

**FIGURE 3:**
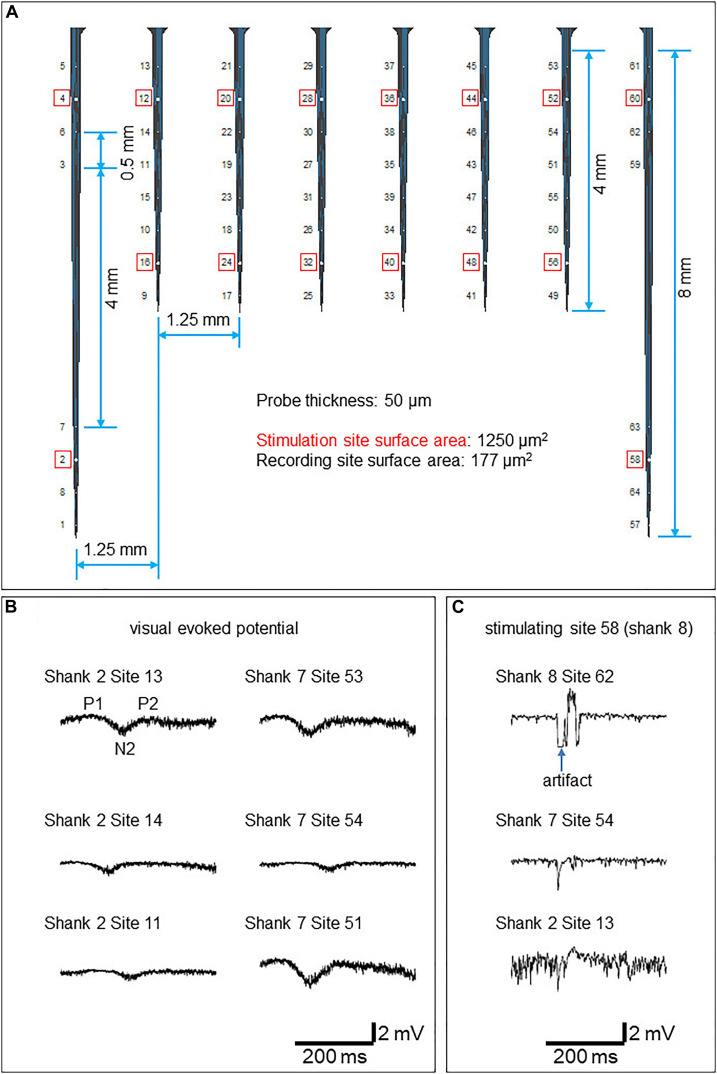
Electrophysiology results **(A)** The probe design. Stimulation sites are circled red. **(B)** Example VEPs recorded from the superficial four layers of electrodes in response to LED flashlight. **(C)** Example evoked responses stimulated by 100 μA current conducted at electrode 58 (shank 8).

**FIGURE 4:**
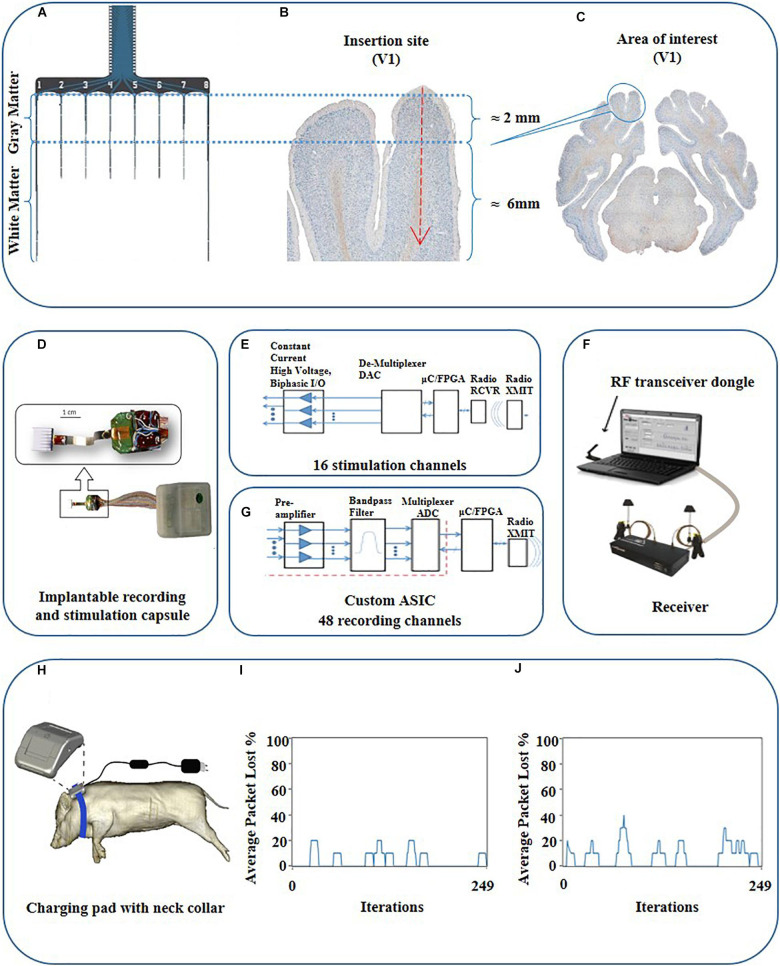
Schematic view of the probe, target area, and schematic description of the communication loop between the PC and the fully implantable wireless BCI device. **(A)** Schematic view of the probe with corresponding parts for gray and white matter. **(B)** Magnified view of the targeted area (V1). **(C)** Histological coronal view of the brain at the level of the visual cortex. **(D)** Implantable combo 16 channel stimulation and 48 channel recording capsule interfaced with 64 electrodes cortical array. **(F)** System overview consisting of RF communication via dongle and receiver to trans-receive commands as well as neural data enabled by independent recording and stimulation software, respectively, along with the implantable capsule with the electrode array. **(E)** Functional diagram highlighting the information flow through various components of the system for stimulation. **(G)** Functional diagram for wireless recording system. The red dots are the whole box from preamplifier to multiplexer ADC, which is the custom ASIC. **(H)** Drawing of near-field induction charging pad worn as a neck belt around the pig. **(I)** Radio-communication loss for phantom gelatin 2 cm thick to simulate animal tissue with a loss of 4% and **(J)** Radio loss of 7% in air.

For achieving the methodological milestone to record and manipulate cortical gray and white matter activity with real-time and closed-loop communication ability, we implemented an integrated telemetric system including a custom-built 8-shank multielectrode probe. The fully implantable array consists of 16 independent electrical stimulation channels and 48 channels for continuous recording (Figures 3A, 4A,D). The implant was designed to record local field potentials (LFP) and multi-unit activity (MUA). The main components of the system entailed an electrode interface, custom application-specific integrated circuits (ASICs) (Figures 4E,G), wireless radiofrequency communication (RF) (Figure 4F), hermetic packaging capsule (size = 70 × 50 × 15 mm, Figure 4D), computer software for bidirectional communication with the implantable device and induction charging pad to be worn around the pig’s neck like a collar (Figures 4D–H).

We have previously reported a former version of this technology (Deshmukh et al., 2020). The interfacing targets had been peripheral neural interfaces which are easier to interface with an implanted capsule. Here, we introduce the technology to be implanted intracerebrally, a decisive new step in terms of technology development. In detail, these were the technical changes and improvements: I. The electrode here represents a multichannel stimulation and recording array. II. In this publication, we developed a 16-stimulation channel and 48-recording channel integrated into the same capsule. III. To provide the power for operating the system we implanted the wireless inductive chargeable system and battery, and IV. to cater to size restrictions, we redesigned our PCBs to lower current requirements. The current draw is reduced from 60 mA to 45 mA.

### Electrode Interface

A custom intracortical electrode array (NeuroNexus, Ann Arbor, MI, United States) consisting of 64 planar silicon electrodes distributed on 8 shanks was designed to study the spatial extent of gray and white matter excitability in the visual cortex. Two electrode types were designed; the stimulatory electrodes to target the white matter and gray matter, respectively, and the recording electrodes (Figure 4A). The total length of the array was 10 mm. Each shank has ventral (deeper) and dorsal (closer to the surface) groups of electrodes consisting of stimulation and recording channels for each white matter and the overlying cortex (Figures 3A, 4A). The surface area of the stimulatory electrodes was 1250 μm^2^ while the area of the recording electrodes was 177 μm^2^. The electrodes were separated by 500 μm, except for the 4th and 5th electrodes from the upper side on shanks one and eight which were separated by 4 mm to record deeper in white matter. The electrode was attached to the telemetric device using a 64 channel Omnetics connector and wires coated in silicon.

### Electrophysiology Protocol

Data were recorded at a 27.7 kHz sampling rate. For stimulation, biphasic constant current pulses (*f* = 0.2 Hz; pulse duration: 0.1 ms/polarity) were conducted at one of the stimulation electrodes at a given time. To probe local excitability in gray matter, 5–30 μA current was administered at dorsal stimulation electrodes 4, 12, 20, 28, 36, 44, 52, and 60. To probe white matter integrity, 100–400 μA current was administered at ventral stimulation electrodes 2, 16, 24, 32, 40, 48, 56, and 58 (Figure 3A). To ensure the physiological origin of the recorded data, visual evoked potentials (VEPs) were recorded in response to a LED flashlight at the right eye in a dimly illuminated room. For electrical stimulation, biphasic constant current pulses (*f* = 0.2 Hz; pulse duration: 0.1 ms/polarity) were conducted at one of the stimulation electrodes at a given time.

### Histology

Three months after irradiation, the animal was humanly killed by an overdose of Pentobarbital (400 mg/ml) and transcardially perfused with 5 liters of 10% buffered formalin (Ettrup et al., 2011). The brain was then removed and post-fixed for 5 days in the same fixative and afterward sliced into 1.25 cm thick coronal tissue slabs (Sorensen et al., 2000). The tissue slabs containing areas of interest were cryoprotected in a 30% sucrose solution in buffered saline (PBS) for 10 days, followed by freezing in isopentane cooled by dry ice. The frozen brain slabs were then cryostat sectioned into 40 μm thick sections, which were either directly mounted on the microscopic slides or preserved free-floating in DeOlmos cryoprotecting solution. The response of the brain tissue was visualized using Nissl, anti-MBP, anti-GFAP, and isolectin stainings.

## Results

### Recording System

A low noise, mixed-signal preamplifier, and multiplexing ASICs for 48 input channels was developed for this study. These small printed circuit board (PCB) electronics encapsulate the ASICs for low noise pre-amplifiers and bandpass filters (Won et al., 2002; Obeid et al., 2003) along with the harvester circuits, RF voltage controlled oscillator (VCO), transmitter power amplifier, and antennas. Harvester circuits detect the wireless fluctuating magnetic fields of the charging pads and convert this magnetic energy into an electrical voltage to operate the circuit boards.

The maximum data sampling rate is 50 kHz with a signal bandwidth of 0.8 Hz to 7 kHz. The custom wireless radio has a wideband frequency modulation (FM) architecture for low power and high data rates, consisting of a radiofrequency (RF) voltage controlled oscillator (VCO), RF amplifier, and splitter to two chip antennas. The receiver consists of an RF demodulator and analog demultiplexer which feeds the data into a 16-bit data acquisition (DAQ) system, or to raw analog signal outputs. Input referred noise was ∼5 uV/root Hz. The frequency of operation for the wireless recording system was 2.7 GHz. The analog data may be viewed simultaneously for all channels in the custom software (“Neuroware”). The software also marks event timestamps regarding behavioral activity or precise stimulation times with less than 1 ms delay in the recorded data. The software includes filters that can be applied for viewing LFPs and multi-units with highpass, lowpass, or bandpass filtering. The system technically is capable of SU recording with a programmable bandpass filter, however, we did not use this possibility in this experimental setup. The real-time RF signal strengths of the radio communication were remotely monitored before and during the experiment to ensure its smooth running. The recording system was also synchronized with the stimulation system to automatically record stimulation pattern indicator pulses indicating the start and end of the stimulation (Deshmukh et al., 2020).

### Stimulation System

For stimulation, the system consists of 16 independent channels out of which, user can multiplex between any two constant current output channels with a maximum output of ±4 mA on each channel. Thus at the given time, the user can simultaneously select any two channels from the available 16 channels for a given stimulation pattern by swapping between the two channels but not all 16 at the same time. The minimum multiplexer switching time delay is 20 μs for stimulation channels, which is the fastest the stimulation driver can switch between two selected channels for stimulation patterns. The stimulator radio system consists of an RF transceiver dongle connected to a PC via a USB cable which sends commands to the WiFi chipset within the capsule. The RF transceiver dongle can monitor and control up to 50 independent implantable stimulator capsules, each with their own unique identifier address for communication, and provides feedback to the stimulation software (“StimWare”) installed on the PC. With a transmission range of up to 3 m, the system provides real-time feedback to the PC for the implantable capsule consisting of the battery voltage, temperature, and stimulation pattern status as well as the strength of the wireless charging field. The stimulation system is also equipped to run automated diagnostics on the implant like battery life, charging state, radio signal strength, and *in vivo* electrode impedances.

### Induction Charging System

A novel inductive charging system was designed to supply wireless energy to the active and passive electronic components of the implantable capsules for operation and to charge the battery for continuous recording and stimulation (Figures 4D,H). It consists of an efficient power harvester design which is used to capture the energy produced by the induction from the time-varying magnetic field. The interface is designed by following IEC 60601-1 and IEC 60601-1-2 (safety and effectiveness of medical electrical equipment) standards. The wireless induction charging system consists of a loosely coupled transmitter and receiver coils. The receiver coil is placed in the implantable capsule along with the power harvester electronics. The system uses near field charging that places the receiver and transmitter coils 5–7.5 cm apart. The maximum depth inside the animal to achieve efficient implant powering via the charging pad was approx. 20 ± 2 mm. Each of the coil’s electrical parameters entailing coil quality (Q), inductance (L), and resistance (R) are carefully tuned at a resonate frequency to maximize the efficiency of the inductive powering system for energy transfer. We employed a rechargeable LiON 200 mAh battery. The battery is rechargeable; however, the run time of the battery depends on a couple of factors e.g., the current draw on the battery from the application being used via simultaneous recording and stimulation which draws up to 32 mA per hour whereas stimulation along draws 17–19 mA depending on the stimulation pattern.

**FIGURE 5:**
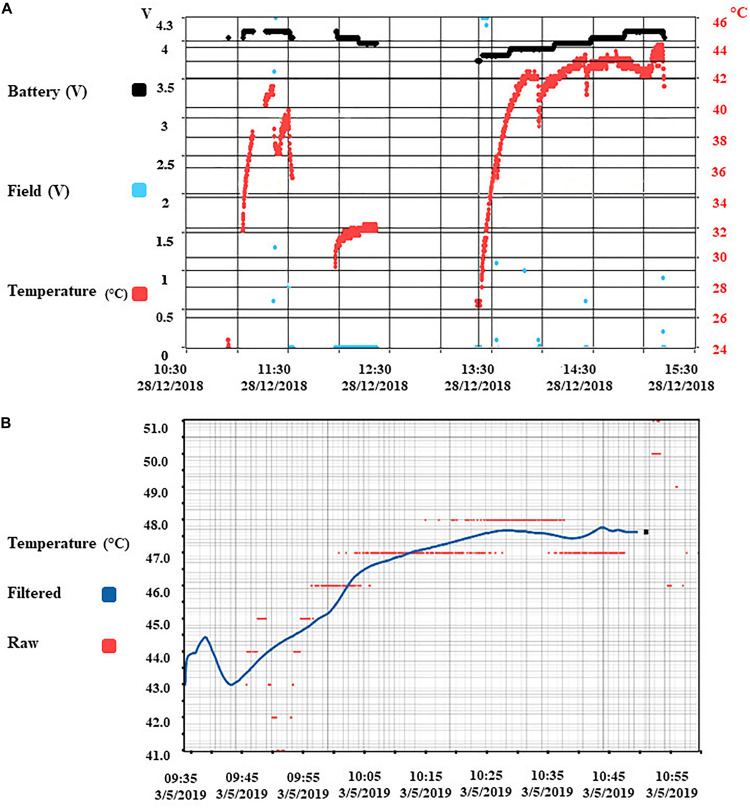
Benchmark results of temperature and radio-communication. **(A)** Core implant temperature profile during wireless charging and full operational use (recording and stimulation). Temperature sensor is located with the electronics inside the capsule. The temperature observed on the shell of the implant capsule is 10 C lower than the core temperature. **(B)** Battery benchmark 3 months post-implantation.

Since the heating of electronics has the potential to cause thermal injury in the implantation’s surrounding tissue, the rise in implant temperature was continuously monitored. The temperature changes due to charging were recorded at three different locations in the lab: Inside the capsule, reporting the temperature value to the software every 2500 ms, at the shell of the capsule via heat sensor strips, and via an infrared camera monitoring the capsule shell and its immediate surroundings. Pre-implantation tests were carried out with the capsule immersed in 38°C water inside an insulated container made up of Styrofoam to prevent heat loss, and monitored over time while in operation for multiple battery life charging cycles. The test was also repeated with the capsule encased inside phantom gelatin to represent body tissue. The outside shell temperature of the capsule was observed to be 8 ± 2 C lower than inside the capsule using infrared cameras. Unfortunately, these tests could not be replicated inside the animal due to the lack of visual observable space in the surgical site. The temperature from inside the capsule during benchtop testing in phantom gelatin recorded a maximum of 44°C (Figure 5A). The battery benchmark 3 months post-implantation was done during the full functional operation, and the core temperature rose to 48 C, which may be estimated at around 38 C on the outer shell (Figure 5B).

### Software User Interface

The stimulation interface was designed so that the stimulation channel alias names would match the electrode site diagram to ease the experimental planning. Sub-channels 1 to 16 each had a unique alias following the format that equates to “Shank number and Electrode Site number” matching the probe design. This enabled the researcher to know exactly which electrode site has been selected for a two-channel simultaneous stimulation from the entire array. The ability to wirelessly switch between 16 channels in real-time over the entire array gives an advanced ability to cover more targets horizontally and along with the depth of the array catering to better spatial resolution for stimulation. The second spatial control is to choose stimulatory electrodes on different shanks (irradiated vs. non-irradiated) that helps the user control the protocol in real-time. The recording channels are configured with a similar format to follow an index of 1 through 52 to identify which electrode site on a particular shank is used from the record channel pinout. Within these 52 channels, there were 48 neural recording channels and 4 reference channels.

### Radio Transmission Quality Testing

The implanted transceiver capsule was tested for the quality of its wireless data transmission by measuring the percentage of data packet loss at a maximum distance of 3.6 m through the air and the animal’s tissue. The stimulator radio power loss at 3.6 m distance was validated using 2 cm of phantom gelatin to represent animal tissue with data presented (Figures 4I,J). The total loss of packets in lab tests was 7% for in the air and 4% in 2 cm thick phantom gelatin. This means 4% of all data packets were not able to complete the full-duplex data link shown in Figures 4E,G between the stimulator to the probe inserted in the animal and then back to the receiving PC transceiver dongle (Figure 4F). The wireless radio frequency (RF) communication device was tuned to a specific frequency (2.4 GHz for the stimulation system and 2.7 GHz for the recording system). The impedance of the RF transmitter board and RF receiver were tuned to the said frequency for maximum wireless signal strength which minimizes the data packet loss. Since the efficiency of the frequency changes in different mediums, the minimum power loss of packets reflects the good quality of the wireless data communicated in the intended tissue. The result is supported by the fact that the radio is tuned for better performance in the animal once implanted as opposed to tuning in just air. The recording radio has been optimized to work best once implanted by adjusting the RF antenna strengths to maximum. The tuning was carried out during the assembly of the device by utilizing the attenuation of the RF in phantom gelatin. It was carried out only once and not during the *in vivo* study. This tuning is considered sufficient for long term implantation as it is carried out by fixing the values of the capacitors and impedances to have maximum resonance. This is maintained with the radio and does not get detuned unless the circuit containing the hardware components somehow gets damaged in the case that the capsule is damaged. The latter is controlled by the percentage packet loss test performed after the device was implanted in the animal (Figure 4). This radio tuning method preempts the loss of signal strength due to intervening animal tissue once implanted. The implanted transmitter device also records the presence of radio-signal lock at the receiver, indicating receipt of a strong signal. The effective range of the telemetry system is 3 m.

### Electrophysiology

The visual evoked potentials in response to the LED stimulation are shown in Figure 3B. In comparison to flash VEP in humans (Odom et al., 2016), putative components P1, N2, and P2 were observed. N1 was not apparent.

Electrically evoked responses by 100 μA stimulation conducted at electrode 58 (shank 8) are shown in Figure 3C. A larger artifact was observed at recording sites in the shank where stimulation was conducted.

### Histology

**FIGURE 6:**
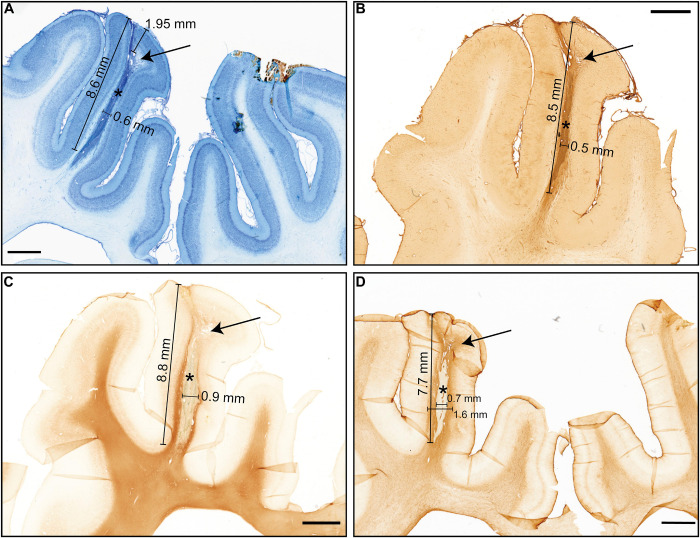
Histology results showing the necrosis and electrode tract with different stainings. The arrows show necrosis and * – electrode tract **(A)** Nissl Staining, black pointer: Edema and necrotic changes, approximate cortex diameter 1.95 mm, **(B)** Isolectin staining (microglia/macrophages), **(C)** Anti-MBP myelin staining pointer: necrotic area, **(D)** Anti-GFAP staining, Tissue with enhanced glia response (Scale bar 2 mm).

The position of the electrode in V1 (visual cortex) was verified using the online atlas of the Göttingen minipig brain (Orlowski et al., 2019). The inflammatory response of the brain tissue around the probe was observed (Figure 6A). The dorsal half, about 2–3 mm, of the visible electrode track, was positioned in the gray matter, the ventral part of the electrode track was situated in the white matter (Figures 6A–D). On the side of the electrode track in the border between cortical gray and white matter, there were visible small irradiation-induced necrotic changes and edema (Figures 6A,B,D). The electrode track visible on the sections is around 0.5 mm wide; the length is around 8 mm (the visible tissue reaction stretches a bit longer – up to 10 mm). The glial scar, with a thickness varying from 0.3 to 0.6 mm, surrounded the electrode track (Figure 6D). According to the cell-morphology, numerous activated microglia cells and macrophages were observed within the small irradiation-induced necrotic area close to the insertion area (Figure 6B).

### Surgical Outcome

One day after BCI implantation surgery, the animal was transported back to the university farm with controlled humidity, temperature, and diet. The wound healed normally during the 10 days after the surgery. While there was no explicit behavior testing, nonetheless, the animal was monitored by the veterinarian nurses on a daily basis, and no signs of obvious behavioral changes, such as increased aggression, or decrease in food intake, had been reported. The animal status was followed for 3 months after the surgery without any sign of infection or failure to thrive.

## Discussion

Assessing neuronal network function while the subject exhibits complex behavioral tasks remains as one of the last methodological frontiers. For a causal understanding of the relevance of the given neuronal ensemble activity for the respective brain function and dysfunction, recording the network signature will not suffice. Therefore, we need to manipulate and modulate the current activity state on the fly, in a closed-loop fashion. BCIs comprising multielectrode arrays would enable bimodal neuromodulatory experimental designs (Grand et al., 2013). Inductive charging and telemetric communication with a fully implanted device will open up new avenues of *in vivo* neuronal activity recordings in a minimally invasive fashion.

We developed this methodological concept that enables us to address the effects of local radiation on the function of neuronal circuits. Even complex and multi-factorial disorders such as depression are thought to be a manifestation of a neuronal circuit disorder, i.e., a dysbalance of the highly interconnected and functionally bound network. It is hypothesized, that the application of stereotactic radiotherapy, even though initially developed for ablation of brain tumors might, at lower doses, lead to neuromodulation (Schneider et al., 2010; Yeh et al., 2020; Zaer, 2020). The study of neuromodulation effects requires identification of the local network signature of spontaneous and sensory-driven activity and the local excitability in a longitudinal fashion to understand the phasic activity of the network and its components. At first, we employed a rather high dose of radiation, known to cause necrosis (Yeh et al., 2020; Zaer, 2020). According to the histology results, the stereotactic radiotherapy was correctly targeted and resulted in irradiation-induced necrosis.

The fully implantable BCI developed here has exhibited stable performance during the study period in terms of the technical specs of the device, such as the battery life, the absence of leakage, quality of radio-communication, and we did not find any significant post mortem inflammatory tissue reaction. The feasibility of inductive charging, wireless recording and stimulation, and the stable signal quality throughout the recording period pioneered a BCI for large animal models. The three-meter-range of the telemetry system permits the experimental subject to behave freely in its environment with minimal interference. Indeed, removing the tethered connections, hypnotic medicine, and the immediate presence of the researcher during the data acquisition should drastically reduce physiological noise and increase data validity. Moreover, the wireless connection will reduce the risk of infection in chronic studies. This fully implantable wireless BCI is capable of providing LFP data, multiunit activities as well as cortical and white matter stimulation on different specified electrodes with simultaneous recording of the real-time responses. Some of the main features of the presented device in comparison to other recently published BCIs are noted in Table 1 (Rouse et al., 2011; Bagheri et al., 2013; Yin et al., 2014; Musk, 2019; Zhou et al., 2019; NeuroPace, 2020; Zhu et al., 2020). Rather than the possibility of wireless inductive charging, other specificities such as high sample rate, ADC resolution, and width bandwidth range can be mentioned as improvements in this study. We perceive it as a strength of our BCI, that it had not been developed as individual components, but in contrast with a comprehensive and unifying goal: the bandwidth, the power of the battery, the software, the impedances of the electrodes for stimulation and recordings had all been tailored for the question at hand, i.e., the recording and stimulation of a neuronal circuit in the visual cortex upon local radio-modulation.

**TABLE 1:** Comparison of different main specifications of recently presented brain-computer interfaces.

Brain-computer interface	Simultaneous Stimulation/recording	Recording channels number	Stimulatory channels number	Wireless induction charging	Sample rate (Hz)	Bandwidth (Hz)	Recording type	ADC resolution (bit)
This study	+	48	16	+	50 kHz	0.7 Hz–8 kHz	LFP-MU	16
WAND (1)	+	128	128	−	1 kHz	500 Hz	LFP	15
Activa PC + S (2)	+	4	8	−	200 Hz	500 Hz	LFP-EEG	10
NeuroPace RNS (3)	+	4	8	−	250 Hz	≥50 kHz	EEG-LFP	10
University of Toronto (4)	+	256	64	−	15 kHz	1 Hz–5 kHz	EEG	8
Neuralink (5)	−	3072	−	−	19.3 kHz	3 Hz–27 kHz	LFP-MU	10
Braingate (6)	−	100	−	−	20 kHz	0.1 Hz–7.8 kHz	LFP-SU	12
WIMAGINE (7)	−	64	−	−	1 kHz	0.5–300 Hz	ECoG	12

*LFP, local field potential; MU, multi-unit activity; SU, single-unit activity; EEG, electroencephalogram; ECoG, electrocorticography (1, 3–6, 8).*

In terms of the closed-loop ability: The system cannot only record but also stimulate, the recorded signals can be displayed and computed in real-time, followed by stimulation. Here, we simply did not showcase an example, which could be, for instance, recording a VEP and stimulating exactly on a determined timestamp after the N1 peak, to probe local excitability.

The wireless communication part of the device can interface with different types of probes according to the study purpose and gives the opportunity of targeting deep brain structures. Other indications of implementing this concept could be probing of deep-brain structures such as the hippocampus, highly relevant for behavioral studies. A large variety of electrode designs from different manufacturers can be connected and integrated into this BCI. Single shank probes with densely spaced recording electrodes for example could even be used for spike sorting, and the identification of putatively inhibitory and excitatory units (Yang et al., 2017). Alternatively, electrode meshes could be used covering large portions of the cortical surface, ideal for studying the propagation of neurophysiological signals, e.g., in the field of epilepsy research. What is more, the capability of simultaneously recording and stimulation opens up closed-loop experimental designs. A key technical advance represents the fully implantable approach and the inductive charging capability, allowing for longitudinal studies. Other examples of the wireless recording of LFP, MU, EOG, EMG together with activity data have also been previously presented, however, lack of closed-loop modulation, inductive wireless charging, and the possibility of full implantation limit their application in neuromodulatory studies (Grand et al., 2013). A major obstacle in the implementation of long-lasting microelectrode-based BCI represents the biological compatibility and degradation of the electrode functionality, mainly due to local gliosis (Lebedev and Nicolelis, 2006; Ball et al., 2009; Orlowski et al., 2017). The second challenging issue is to avoid thermal tissue injury generated by the wireless charging pad. This is intended to be addressed in the future using a variable design of the charging pad belt with variable height adjustments as well as a closed-loop software system. The software system will automatically control the charging duty cycle strength based on data from the temperature and battery life status of the implant, and temperature on the surface of the charging pad.

For BCIs to be implemented in clinical applications in a broader context, it is mandatory to decode network computations from background noise by gaining reproducible, spatially defined signals from specified brain regions (Pfurtscheller et al., 2006). Adequate deciphering is of great importance, providing the necessary input for communication tools, and to at least partially restore motor function in stroke, Locked-in syndrome, and amyotrophic lateral sclerosis patients (Kennedy and Bakay, 1998). Despite advances in developing robotic limbs for plegic patients (Collinger et al., 2013; Bouton et al., 2016) or communication tools for long-suffering Locked-in individuals (Vansteensel et al., 2016), the clinical implementation of these technologies is still extremely challenging. The shortcomings of these systems rely heavily on the current advances in hardware technology. While there is still a long way to go, our proof-of-concept study at least suggests a few key concepts to improve the integrated framework of a highly sensitive and fully implantable BCIs. For mastering the crucial transition from laboratory to in-home BCI use, research efforts should be directed toward enhancing the stability of BCIs concerning user autonomy, long-lasting functionality in terms of permanent availability of neural interfaces with a minimum neural tissue disturbance and irritation (Huggins et al., 2011; Nijboer, 2015).

## Data Availability Statement

The raw data supporting the conclusions of this article will be made available by the authors, without undue reservation.

## Ethics Statement

The animal study was reviewed and approved by the Danish Animal Experiments Inspectorate (2016-15-0201-01103) in compliance with the ARRIVE guidelines and the 2010/63/EU directive for animal experiments.

## Author Contributions

HZ and AD wrote the first draft of the manuscript. HZ, AD, JM, MSJ, JS, P-HP, VG, QF, and AS collaborated on the designing, and benchmarking of the device prototype. HZ and JS did the implantation surgery with the assistance of LF. MBJ, EW, and SL planned and performed the stereotactic radiosurgery together with HZ, JS, DO, and AG. JRA and MBS proposed the concept of the experiment and helped with the study design. DO and TM performed the histology experiment and analysis. P-HP, WF, and AS proposed and performed the electrophysiology protocol and analysis. All authors edited and accepted the final manuscript.

## Conflict of Interest

MBS and JRA are employees of Zap Surgical Systems, Inc., that funded this study, own stock, and have patents in the field of stereotactic radiosurgery without affecting the trial’s outcome. AD, QF, VG, and JM were employees of Triangle BioSystems International as a division of Harvard Bioscience Inc., however, did not have any influence on the result of the experiments. The remaining authors declare that the research was conducted in the absence of any commercial or financial relationships that could be construed as a potential conflict of interest.
